# Evaluating the psychometric properties of the fatigue severity scale using item response theory

**DOI:** 10.1186/s40359-023-01198-z

**Published:** 2023-05-12

**Authors:** Seiji Muranaka, Haruo Fujino, Osamu Imura

**Affiliations:** 1 Graduate School of Human Sciences, Osaka University, 1-2 Yamadaoka, Suita, 565-0871 Osaka Japan; 2 United Graduate School of Child Development, Osaka University, Suita, Japan; 3 Faculty of Social Studies, Nara University, Nara, Japan

**Keywords:** Fatigue, Depression, Item response theory, Graded response model, Stress, Sleep

## Abstract

**Background:**

Fatigue is a common daily experience and a symptom of various disorders. While scholars have discussed the use of the Fatigue Severity Scale (FSS) using item response theory (IRT), the characteristics of the Japanese version are not yet examined. This study evaluated the psychometric properties of the FSS using IRT and assessed its reliability and concurrent validity with a general sample in Japan.

**Methods and measures:**

A total of 1,007 Japanese individuals participated in an online survey, with 692 of them providing valid data. Of these, 125 participants partook in a re-test after approximately 18 days and had their longitudinal data analyzed. In addition, the graded response model (GRM) was used to assess the FSS items’ characteristics.

**Results:**

The GRM’s results recommended using seven items and a 6-point scale. The FSS’s reliability was acceptable. Furthermore, the validity was adequate from the results of correlation and regression analyses. The synchronous effects models demonstrated that the Multidimensional Fatigue Inventory (MFI) enhanced depression, and depression enhanced FSS.

**Conclusion:**

This study suggested that the Japanese version of the FSS should be a 7-item scale with a 6-point response scale. Further investigations may reveal the different aspects of fatigue assessed by the analyzed fatigue measures.

**Supplementary Information:**

The online version contains supplementary material available at 10.1186/s40359-023-01198-z.

## Background

Fatigue is a phenomenon people commonly experience due to daily activity or a medical condition. The prevalence of heightened fatigue is experienced by 20 to 23% of the general population [1]. Fatigue also appears as a common symptom of psychiatric disorders, including depression, anxiety disorders, and sleep disorders [2–5]. However, the prevalence estimates of fatigue in psychiatric conditions vary due to the wide variation in the sample and methodologies (range 10–80%). Samaha et al. [6] found that chronic fatigue has a significant positive correlation with trait anxiety and mood disorder and is correlated with undesirable emotional experiences.

During the COVID-19 pandemic, which occurred in 2020, our way of life was changed; for instance, communication had been transformed into digital-based forms, such as teleconference systems, and people had been exposed to too much information. Teleconferencing can increase fatigue as technical problems arise, which would not occur if we took face-to-face conferences; furthermore, limited information makes us interpret the reactions and expressions of the other participants [7]. In addition, the richness of information can increase event disruption and social media fatigue [8]. Therefore, sustained fatigue should be avoided to prevent mental health issues. Accordingly, validated assessment tools for fatigue severity are essential for research and practice.

The degree of fatigue has been comprehended subjectively or objectively. A few indexes of fatigue include activity amount measured using actigraphy [9], biomarker and neurophysiological response measures [10], performance-based cognitive/behavioral tasks, and subjective assessment using self-report questionnaires [11–13]. Regarding questionnaire scales for subjective fatigue, some examples are the Fatigue Severity Scale (FSS; Krupp et al. [14]) and the Multidimensional Fatigue Inventory (MFI; Smets et al. [15]).

The FSS is a 7-point, 9-item scale measuring fatigue. Raman et al. [16] measured the FSS and brain activity in 58 COVID-19-infected and 30 uninfected participants, finding that COVID-19-infected participants had significantly higher FSS scores than their uninfected counterparts. Sunwoo et al. [17] also examined factors affecting fatigue and defined high fatigue as an FSS score of 4 or more. They reported that having at least three days per week of no physical activity, drinking alcohol at least twice a week, sleeping in for long periods on holidays, being aware of lack of sleep, intense daytime sleepiness, and high depression were risk factors for high fatigue. The MFI is a 5-point, 20-item questionnaire with five factors. Morin et al. [18] used the MFI as one of the validity indices in an Insomnia Severity Index (ISI) scale development study to measure insomnia severity and reported significant positive correlations between the ISI and each of the five factors of the MFI. In summary, the fatigue scales developed in previous studies have helped investigate the correlations between fatigue and infectious, physical, and psychiatric illnesses.

However, the psychometric properties of the FSS remain a controversial topic. Lerdal and Kottorp [19] noted that the 7-item FSS (FSS-7, excluding Items 1 and 2) has higher reliability and validity and may be more sensitive to changes in fatigue. They also pointed out that the FSS-7 may have higher reliability and validity in measuring the degree of interference due to fatigue rather than fatigue severity [20, 21]. In the validation process, concurrent validity should be evaluated as a measure of its characteristics by assessing the associations with related concepts (e.g., depression, sleep, and stress). Accordingly, the measurement performance of the FSS should be confirmed in Japan and then examined for future use.

Subjective measurements such as patient-reported outcomes (PROs), which include fatigue, were evaluated with several approaches: classical test theory (CTT), item response theory (IRT), and Rasch measurement theory (RMT). Each approach has pros and cons, each evaluated by Petrillo et al. [22], who pointed out four weaknesses of CTT. The first is the difficulty of the level of scale: item-level data are based on ordered counts, but CTT evaluations imply interval-level measurement. Second, CTT results depend on the interaction between sample and scale properties, which leads to serious logical drawbacks. Third is the difficulty of handling missing data. Finally, the standard measurement error around individual patients’ scores is assumed to be a constant value regardless of the person’s location on the scale range. Therefore, modern approaches (i.e., IRT and RMT) were recommended for evaluating psychological measurement because they can evaluate it with weaker sample, scale, and distribution restrictions.

IRT was proposed and often used in psychology and educational studies. It predicts the latent trait value of the respondent and evaluates the measurement accuracy from the consistency between the latent trait value and the actual measurement value [23, 24]. The Rasch [25] model is an IRT method to estimate the accuracy of a questionnaire scale by predicting the difficulty of responding to an item according to a respondent’s ability. It can be used to evaluate binary scales; the Rasch rating-scale model has also been extended to predict the difficulty at each stage of a Likert scale with three or more items [26]. Lerdal and Kottorp [19] evaluated the measurement performance of the FSS using the Rasch model and found that the first and second items of the FSS had high outfit Mean Square (MnSq) Statistics value, and the average step calibration of the second item did not advance monotonically; thus, they proposed a 7-item FSS excluding these items.

Another IRT for multilevel scales is the graded response model (GRM; Samejima [27]), which takes a respondent’s ability $\theta$ as an input and gives the category m and the response probability ${P}_{m}$ using the following equation:

$${P}_{m}\left(\theta \right) = {{P}^{*}}_{m}\left(\theta \right)-{{P}^{*}}_{(m+1)}\left(\theta \right)$$

$${{P}^{*}}_{m}\left(\theta \right)=\frac{1}{1+exp\left(\alpha \right(\theta -{b}_{m}\left)\right)}$$

For a given item, the GRM predicts the difficulty of responding to a category larger than the specific response category corresponding to the respondent’s ability. Thus, compared with the Rasch model and its multiple-stage application, the GRM considers the ordinal relationship among categories; considering these characteristics, we deemed it appropriate to use GRM—over the Rasch model, which has been used in previous studies (e.g., Lerdal and Kottorp [19])—for evaluating the FSS.

This study aimed to assess the psychometric properties of the FSS using IRT analysis and its reliability and concurrent validity with a general Japanese sample. The validity of the FSS was assessed in relation to another fatigue measure (MFI), depression, sleepiness, and stress because these relationships were pointed out by Sunwoo et al. [17]; Lerdal et al. [21] assessed the validity of FSS using daytime sleepiness.

## Materials and methods

### Participants

The study was conducted between February and March 2021 (February 22 to March 12, 2021). A total of 1,007 participants who were not receiving treatment for mental or physical illnesses and had no cognitive problems by self-report participated in the study. Participants were balanced by 10 (5 age x 2 sex) blocks, which were divided into 10 years from the twenties to the sixties and sex. The online survey system recorded the duration participants responded to the questionnaire. We excluded participants who responded within 3.5 min while considering the number of survey items. Data from 692 participants (age: mean = 47.03, SD = 12.75; 328 male, 364 female) were considered to be valid and used in the subsequent analyses. The distribution of sex and age structure in the current sample was not substantially different from the Japanese population census in 2020 (https://www.stat.go.jp/english/data/kokusei/index.html). A second survey was conducted 18 days after the first survey (March 11–12, 2021), which yielded valid data for 125 individuals, corresponding to those from the first survey.

### Procedure

This survey was conducted with the approval of the Research Ethics Committee of the Faculty of Social Studies, Nara University (ID: 2020-5-2). An online survey was conducted by Cross Marketing Inc., a research company that crowdsources survey participation from registered users. Participants in the survey responded to the following questions: demographics (e.g., age, sex, and occupation), MFI, FSS, Patient Health Questionnaire-9 (PHQ-9), sleep duration, and stress level at work or school. Research participants who responded to all questionnaire items were rewarded with an amount of money stipulated by the research company.

### Measures

*Multidimensional Fatigue Inventory*. Participants were asked to complete the Japanese version of the MFI [15, 28]. The MFI was developed to measure the degree of fatigue according to five dimensions: general fatigue, physical fatigue, reduced activation, reduced motivation, and mental fatigue. The MFI has been widely used and validated in various populations and countries, including Japan. A total of 20 items, four for each dimension, are scored on a 5-point scale where one is “no, that is not true at all,” and five is “yes, that is completely true.” The reliability of the Japanese version of the questionnaire was deemed acceptable [28].

*Fatigue Severity Scale*. The study participants were asked to respond to the FSS [14], a 1-factor, 9-item measure of fatigue. Although originally developed for clinical groups such as patients with multiple sclerosis, the FSS has also been used in the general population [29, 30]. Respondents answer items using a 7-point scale where one is “completely disagree,” and seven is “completely agree.”

*Patient Health Questionnaire*. The participants were asked to respond to a 9-item scale developed by Spitzer et al. [31] to screen for depression in primary care. The Japanese version of this scale was validated by Muramatsu et al. [32]. The PHQ-9 is used in many countries to screen for depression and assess its severity. In this study, the measure was used as an index to assess depression severity [33]. For each question, respondents were asked to indicate the frequency with which they were bothered by symptoms in the past two weeks using a 4-point scale, ranging from 0 for “not at all” to 3 for “nearly every day.”

*Sleep duration*. The study participants were asked to select their average nightly sleep duration for the previous week using seven hourly discretized options ranging from “less than 4 hours” to “more than 9 hours.”

*Stress in the work or school environment*. The participants were asked to describe their work or school environment using one of the following options: mentally stressful, physically stressful, mentally and physically stressful, or not very stressful.

### Statistical analysis

We used GRM IRT to evaluate the measurement accuracy of the FSS. First, to confirm the assumption of the analysis, the unidimensionality of the original FSS was checked using factor analysis, and then the item parameters were estimated. Subsequently, the item characteristic curve (ICC), item information curve (IIC), and test information function (TIF) were examined. In this study, for ICC, the horizontal axis is the parameter indicating fatigue intensity, and the vertical axis is the reaction probability of the response categories with respect to fatigue intensity. If the peaks of the reaction probabilities appear in an order based on fatigue intensity, the item can be evaluated as measuring the fatigue aspect well. For IIC, the horizontal axis indicates fatigue intensity, and the vertical axis is the amount of information in each item. The TIF is a plot of the sum of the IICs of each item, which allows us to evaluate the characteristics of the whole scale.

We first examined the ICC, IIC, and TIF of the original 9-item, 7-point response scale, and then also similarly examined (1) models that removed items with limited information based on the IIC, (2) integrated grades that could not distinguish the rating grades based on the distribution of the ICC, and (3) models that implemented both of these (1 and 2). After evaluating the properties of the FSS as a measurement scale, we determined the final use of the FSS.

Regarding the FSS score calculation methods, we examined the differences between the FSS scores calculated according to the following methods by correlation: (a) using the original score calculation method (i.e., mean of all item scores) with the items selected according to IRT analysis; (b) using the IRT-estimated coefficients with the items selected according to IRT analysis. The calculation of the FSS score in this study was determined based on the above analysis. The correlations between the original FSS scores using the nine items and the FSS scores calculated by IRT analysis in this study were also reported for comparison with the original method.

Second, demographic statistics of the participants and descriptive statistics of the FSS, MFI, PHQ-9 were described; for the FSS, scores calculated using the original method and IRT of the present study were reported as reference values to compare with previous studies. Moreover, the intraclass correlation was calculated to assess the test-retest reliability using data from the first and second survey applications (n = 125). Pearson’s correlation coefficients were also calculated and evaluated for correlations between the FSS and other measured scores to examine the properties of the FSS scores selected based on item characteristics using IRT.

Third, we compared the results of the FSS based on IRT and the MFI, which has already been widely used as a validated fatigue measure in Japan and examined the validity of the FSS constructed based on the IRT. A one-way ANOVA was conducted to investigate the association between fatigue and the description of how stressful work or school environments are and between fatigue and sleep duration. Tukey’s honestly significant difference test was used for multiple comparisons between groups. Those who reported their occupation as unemployed at the time of the survey were excluded from the analysis of stress in work or school environments. Pearson’s correlation coefficients were used to assess the correlation between MFI, FSS, and depression.

Finally, differences in characteristics between the FSS and MFI were examined. First, the relationship between fatigue and depression was confirmed using correlation analysis. Second, the relationship between fatigue and depression was examined using multiple regression analysis. In addition, using the data from the first and second surveys, we constructed a cross-lagged effects model and synchronous effects model to investigate the longitudinal effect of the FSS or MFI on depression. These two models were constructed using measurements from two-time points. The cross-lagged effects model was designed to compare the effects of two variables from the Time 1 variable on the Time 2 variable; Berry and Willoughby [34]). The synchronous effects model is a better fit when the measurement interval between Time 1 and Time 2 is longer [35].

The significance level for statistical hypothesis testing was set at 5%. The above analyses were performed using R (ver. 4.1.2). The ltm package (ver. 1.2.0 [36]) was installed for IRT implementation.

## Results

### IRT of the FSS

First, the GRM IRT was conducted using the 9-item FSS. As a result of confirming the scree plot, the eigenvalues were found to transition between 5.85, 1.18, 0.44, and 0.39, which were considered unidimensionality. After estimating the number of item parameters, the ICC, IIC, and TIF of each item were confirmed.

The results of the ICC showed that the response probability of Grade 7 increased before the peak of the response probability of Grade 6 for all items, and the difference between Grades 6 and 7 did not reflect a high level of fatigue. In particular, in Items 1 and 2, the reaction probabilities of the grades did not peak with respect to fatigue intensity, and it was confirmed that the dispersion was large. Items 1 and 2 had little information about the degree of fatigue. Item 3 was also found to have a large amount of information even when fatigue was relatively weak, and the others were more responsive when fatigue was moderate to strong. The IIC results revealed that the information quantity of Items 1 and 2 was uniformly low in relation to fatigue intensity. Item 3 had less information quantity than the other items except for Items 1 and 2.

Based on these results, three additional conditions were considered: (1) remove Items 1 and 2; (2) integrate Grades 6 and 7; (3) do both (1) and (2). In all conditions, unidimensionality was confirmed. In Condition 1, the issue of Grades 6 and 7, which could not be distinguished, remained similar to the result of the ICC. In Condition 2, the IIC indicated that the information quantity of Items 1 and 2 was uniformly distributed with respect to fatigue intensity (Figures S2 and S3). Furthermore, only Condition 1 had a lower TIF than the other conditions.

**Fig. 1:**
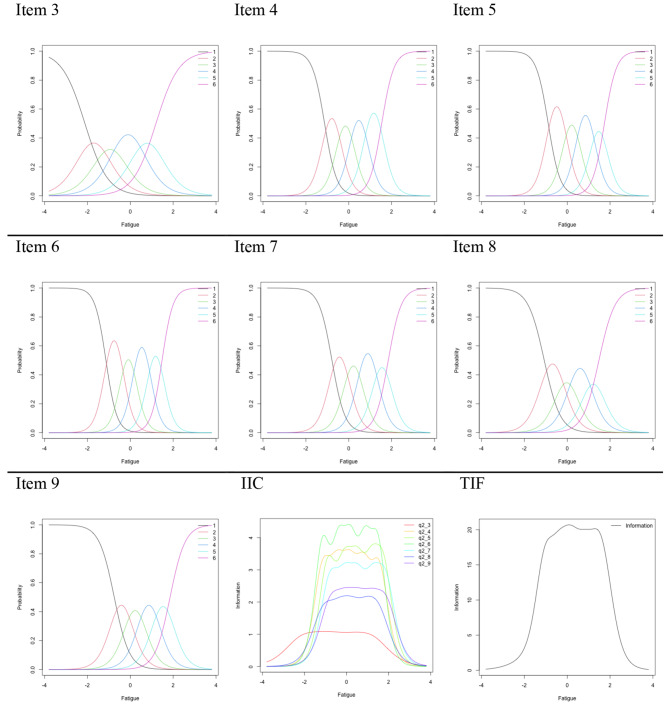
Item characteristic curve, item information curve, test information function of Condition 3. In Condition 3, Items 1 and 2 were removed and Grades 6 and 7 were integrated. ICC: Item characteristic curve, IIC: Item information curve, TIF: Test information function

The ICC, IIC, and TIF for Condition 3 are shown in Fig. 1. From the ICC, there was a correspondence between grade response probability and fatigue intensity, and the IIC indicated that information quantity increased in specific areas of fatigue intensity. Regarding TIF, no decrease was seen when the condition was set as six levels of seven items from the original FSS items, and the loss of information quantity was limited. Therefore, the results reported below were achieved using the FSS with Condition 3 (i.e., Items 1 and 2 were removed, and Grades 6 and 7 were merged).

The correlation coefficient between the scores calculated by averaging each item of those selected by the IRT and the scores calculated by using the coefficient of difficulty of each item of those selected by the IRT was very high (*r* = .99, *p* < .001 [from fss x fss_irt]). As the scores were almost identical when the mean of the items was used to calculate the scores, the FSS score (FSS [IRT]) was calculated from the mean of the 7-item, 6-point scale selected by IRT for the convenience of the scale in the survey. The correlation between the original FSS and the FSS (IRT) was high (*r* = .97, *p* < .001).

### Descriptive statistics

Table 1 presents the characteristics of the 692 participants. The descriptive statistics of the scales are shown in Table 2. The intraclass correlations for all scales were high; however, the FSS (IRT) was relatively low at 0.59.

**Table 1:** Participants’ characteristics

Variable	n
Sex: female/male	364/328
Age	
20–29	110
30–39	122
40–49	133
50–59	158
60–69	169
Working status	
Working	623
Stress in the work or school environment	
Mental	176
Physical	47
Mental and physical	93
None	307
Average sleep duration	
Less than 4 h	16
4–5 h	79
5–6 h	189
6–7 h	198
7–8 h	110
8–9 h	24
9 h or more	7

Note. N = 692

**Table 2:** Descriptive statistics

	Mean^a^	SD	Skewness	Kurtosis	Cronbach’s alpha	ICC^b^	95% CI lower	95% CI higher
MFI	55.73	12.75	-0.07	0.09	0.91	0.85^***^	0.79	0.89
General fatigue	11.95	3.36	-0.03	-0.17	0.80	0.79^***^	0.72	0.85
Physical fatigue	10.93	3.35	0.10	-0.11	0.80	0.79^***^	0.72	0.85
Reduced activation	10.70	3.17	0.23	-0.02	0.69	0.76^***^	0.68	0.83
Reduced motivation	11.06	2.81	-0.07	-0.02	0.53	0.74^***^	0.65	0.81
Mental fatigue	11.10	2.79	0.04	0.54	0.66	0.71^***^	0.61	0.79
FSS (Original)	3.52	1.30	0.20	-0.20	0.93	0.62^***^	0.50	0.71
FSS (IRT)	3.22	1.31	0.10	-0.74	0.94	0.59^***^	0.46	0.69
PHQ-9	4.75	5.21	1.71	3.56	0.91	0.83^***^	0.77	0.88

Note. N = 692. 95% CI: 95% confidence interval, FSS: Fatigue Severity Scale, FSS (IRT): Fatigue Severity Scale (Item Response Theory), ICC: Intraclass correlation, MFI: Multidimensional Fatigue Inventory, PHQ-9: Patient Health Questionnaire-9, SD: Standard deviation

^a^ participants who responded to the first survey (N = 692)

^b^ participants who responded to both surveys and had their longitudinal data analyzed (n = 125)

^***^p < .001

### Relationship between stress situation and fatigue

A one-way ANOVA was conducted to examine the relationship between mental and physical stress situations and fatigue. The independent variables were the presence of mental and physical stress conditions. The results are shown in Table 2. Multiple comparisons (Tukey’s honestly significant difference test) were also conducted for variables found to be significant in the ANOVA results, and 95% CIs are shown in Table 3.

For the MFI, the ANOVA results were significant (*F* [3, 619] = 21.14, *p* < .001, *Cohen’s f* = 0.32). Multiple comparisons revealed that Group 4 (environment without much stress) was significantly lower (*p*-values < 0.001) than Group 1 (mentally stressful environment) and Group 3 (mentally and physically stressful environment).

The one-way ANOVA results were significant for the FSS (IRT) (*F* (3, 619) = 19.84, *p* < .001, *Cohen’s f* = 0.31). Multiple comparisons demonstrated that Group 4 was lower than Group 1 and Group 3 (*p*-values < 0.001); thus, the same groups were found to have significant differences in the results for both the MFI and the FSS (IRT).

**Table 3:** Multiple comparison of the Multidimensional Fatigue Inventory and the Fatigue Severity Scale (Item Response Theory) by environmental stress status groups

		Group 1 (n = 176)	Group 2 (n = 47)	Group 3 (n = 93)	Group 4 (n = 307)	Multiple comparison by Tukey’s honestly significant difference [95% CI]
MFI	Mean	59.32	55.72	59.69	51.52	4 < 1 [-10.71, -4.9] 4 < 3 [-11.81, -4.53]
	SD	(11.75)	(8.85)	(11.39)	(12.53)	
FSS (IRT)	Mean	3.56	3.29	3.74	2.83	4 < 1 [-1.03, -0.43] 4 < 3 [-1.29, -0.53]
	SD	(1.25)	(1.13)	(1.25)	(1.25)	

Note. N = 623. FSS (IRT): Fatigue Severity Scale (Item Response Theory), MFI: Multidimensional Fatigue Inventory, SD: Standard deviation

### Relationship between sleep duration and fatigue

**Fig. 2:**
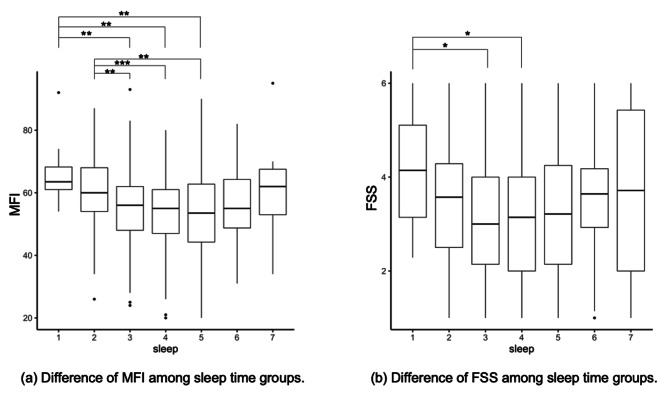
Boxplot of the Multidimensional Fatigue Inventory and the Fatigue Severity Scale by sleep duration group. MFI: Multidimensional Fatigue Inventory, FSS: Fatigue Severity Scale. Legend for sleep: 1, less than 4 h; 2, 4–5 h; 3, 5–6 h; 7, 9 h or more. The sleep duration indicated on the right side of the hyphen is not included in the group but is included in the next group (i.e., Group 2 included the persons who had 4 or more and less than 5 h of sleep). * *p* < .05, ** *p* < .01, *** *p* < .001

A one-way ANOVA was conducted to examine the relationship between average weekly sleep duration and fatigue. The independent variable was a 7-level categorical variable in which the average hours of sleep per week were discretized into one-hour units, ranging from “less than 4 hours” to “9 hours or more.” The relationship between MFI and FSS (IRT) scores and sleep duration is visualized in Fig. 2. For the MFI, Group 1 (less than 4 h) and Group 7 (9 h or more) revealed a gradual U-shaped transition with higher scores.

For the MFI, ANOVA results were found to be significant (*F* [6, 616] = 5.862, *p* < .001, *Cohen’s f* = 0.17). Multiple comparisons revealed that Group 1 (less than 4 h) and Group 2 (4–5 h) were significantly higher (*p*-values < 0.01, 95% Confidence Intervals [CIs] = [-22.02 -1.48]) than Groups 3, 4, and 5 (6–8 h). The results for the FSS showed a U-shaped curve similar to that for the MFI. ANOVA results were significant (*F* [6, 616] = 2.87, *p* < .01, *Cohen’s f* = 0.17). Multiple comparisons established that Group 1 was higher than Groups 3 and 4 (*p*-values < 0.05).

### Correlation analysis

The FSS (IRT) and MFI indicated moderate correlations (*r* = .62, *p* < .001) with each other, and with the PHQ-9 (MFI: *r* = .62, *p* < .001; FSS [IRT]: *r* = .52, *p* < .001).

### Regression analysis

Multiple regression analysis was conducted with PHQ-9 as the dependent variable and the two fatigue scales as independent variables. The adjusted *R*^*2*^ of this model was 0.410. The standardized partial regression coefficients were significantly higher for the MFI (*β* = 0.479, *t* = 12.83, 95% CI=[0.406 0.553], *p* < .001) and FSS (IRT) (*β* = 0.222, *t* = 5.94, 95% CI=[0.149 0.295], *p* < .001), and those for the MFI were higher than those for the FSS (IRT). The variance inflation factor (VIF) was 1.63.

### Association between fatigue and depression using a cross-lagged effects model

A cross-lagged effects model was conducted to test the time-series, pre- and post-temporal relationship between fatigue and depression (Figure S5) using data from 125 individuals with correspondence at two-time points. In the MFI and PHQ-9 (Figure S5a), both the FSS (IRT) and PHQ-9 (Figure S5b) were saturated models. For the MFI, there was a significant positive effect from the Time 1 MFI on Time 2 PHQ-9 (*β* = 0.251, *p* < .001); the error covariance was also significantly higher. A significant positive effect from FSS (IRT) was identified on PHQ-9 of Time 2 (*β* = 0.116, *p* < .05); error covariance was also significantly higher.

### Association between fatigue and depression using a synchronous effect model

**Fig. 3:**
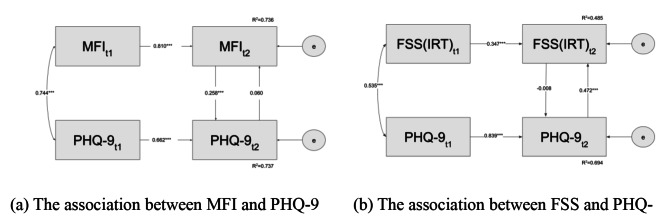
Results for the synchronous effects model. Note. N = 125. MFI: Multidimensional Fatigue Inventory, FSS: Fatigue Severity Scale, PHQ-9: Patient Health Questionnaire, t1: Time 1, t2: Time 2. *** *p* < .001

In the cross-lagged effects model, the error covariance was significantly higher. Therefore, we can consider the possibility that the variables in Time 2 are significantly affected by variables other than those specified in Time (1) One of the factors is that the measurement interval between Time 1 and Time 2 is approximately 18 days. That is, the period may be spread too far apart to explain the high variability of Time (2) We, therefore, also examined the synchronous effects model (Fig. 3).

For the MFI and PHQ-9 (Fig. 3a), the goodness-of-fit indices were acceptable (*χ*^*2*^(1) = 1.907, *n.s.*, *GFI* = 0.992, *AGFI* = 0.922, *CFI* = 0.998, *RMSEA* = 0.085), confirming a significant positive effect from Time 2 MFI on Time 2 PHQ-9 (*β* = 0.258, *p* < .001). Furthermore, the effect from Time 2 PHQ-9 on Time 2 MFI was found not to be significant (*β* = 0.060, *n.s.*).

A similar examination was also conducted for the FSS (IRT) (Fig. 3b). The goodness-of-fit indices were not acceptable (*χ*^*2*^(1) = 5.259, *p* < .05, *GFI* = 0.979, *AGFI* = 0.793, *CFI* = 0.985, *RMSEA* = 0.185), indicating that the model may not fit the data. The effect of Time 2 FSS (IRT) on Time 2 PHQ-9 was not significant (*β* = -0.008, *n.s.*). However, a significant positive effect from Time 2 PHQ-9 on Time 2 FSS (IRT) was identified (*β* = 0.472, *p* < .001).

## Discussion

### Evaluation of the FSS measurement performance by IRT

This study assessed the psychometric properties of the FSS using IRT analysis and evaluated its reliability and concurrent validity with a general Japanese sample. Our IRT results for the FSS, similar to the findings of Lerdal and Kottorp [19] and Johansson et al. [20], indicated that using the FSS as a 7-item scale (after removing Items 1 and 2) may be better to measure fatigue severity. The ICC results demonstrated that neither the frequency of responses to Items 1 and 2 nor the information quantity increased according to fatigue severity. Lerdal et al. [21] also recommended the use of a 7-item FSS without Items 1 and 2 in their measurement of the FSS for HIV-infected individuals; this recommendation stemmed from the mean step calibration not advancing monotonically and the outfit MnSq having values higher than acceptable. The current study, using different models and samples, supports the conclusion that removing Items 1 and 2 is expected to improve the measurement performance of the FSS. Thus, using seven items in the FSS is desirable, even if the GRM is used for survey data of general samples. However, when the number of items was set to seven (Condition 1), the information quantity presented in the TIF was reduced compared to the other conditions, thereby suggesting the need to exclude items and modify the rating scale of the FSS.

Furthermore, the results of the IRT in this study recommended using a combined Grades 6 and 7 scale. The results of the IRT against the original FSS showed that the IIC was biased toward the right regarding increased information quantity, while the scale with 6 Grades and seven items (FSS [IRT]) showed almost symmetrical results. This result suggests that the original FSS had scale characteristics that tended to bias the responses toward those with high fatigue, whereas the FSS (IRT) improved the information bias. Thus, the items and number of steps selected by the IRT led to desirable scale properties for assessing fatigue.

To confirm the validity of the FSS (IRT), we examined correlations between the MFI and factors related to fatigue. The FSS (IRT) correlated well with the MFI, and both correlated moderately to highly with depression severity. The intergroup differences in the influence of environmental stress on the FSS were similar to those on the MFI. These results indicate the validity of the scale for measuring fatigue. In relation to sleep, the differences between groups detected by the FSS (IRT) were consistent with those by the MFI. However, the MFI showed some intergroup differences that were not detected by the FSS (IRT). This may indicate that the MFI is more useful for detecting small differences in fatigue by sleep duration.

Furthermore, fatigue was found to have a U-shaped relationship with sleep duration, implying that shorter or longer sleep duration was associated with the experience of higher fatigue. Sunwoo et al. [17] conducted a questionnaire survey among Koreans with an average age of 47.9 years and found that those who slept for less than 6 h reported higher FSS scores than those who slept for more than 6 h. The mean age in the present study was similar, but the boundary of sleep duration that produces high fatigue was different; however, the fact that the study was conducted with a Japanese sample might account for this difference. Scholars could continue to examine the correlation among fatigue, sleep duration, and cultural differences in future studies.

### Correlation between fatigue and depression

The results of the correlation analysis established that the PHQ-9 significantly correlated with the FSS (IRT) and the MFI. In the regression analysis, the degrees of both FSS (IRT) and MFI were significantly enhanced by the PHQ-9 point, with the regression coefficient for PHQ-9 being stronger with the MFI than the FSS (IRT). The results suggest that the MFI may be preferable over the FSS (IRT) for examining the general sample’s association between mental health and fatigue.

Furthermore, an examination using cross-lagged and synchronous effects models showed that the PHQ-9 enhanced the FSS (IRT), while the MFI enhanced the PHQ-9. This difference in the pre- and post-relationship between the MFI and PHQ-9 on the FSS (IRT) suggests that the MFI and FSS (IRT) may be measuring different aspects of fatigue. Regarding the MFI, Dirzyte et al. [37] examined the relationship between e-learning and mental health in a general sample and indicated the possibility that fatigue measured by the MFI enhanced the depression results. It also suggests that the MFI may measure the depression-enhancing aspect of fatigue. As the FSS-7 (excluding Items 1 and 2) implies the possibility that it has high reliability and validity in measuring the interference level in one’s life due to fatigue rather than fatigue severity [20, 21], the FSS-7 may reflect a correlation between increased depressive symptoms and increased interference of fatigue in one’s life. These characteristics of the FSS may explain the difference between the MFI and FSS results observed in this study.

However, the FSS (IRT) proposed in this study has a different response scale (i.e., a 6-point scale) than the traditional FSS and the FSS-7. In addition, the model describing the association between depression and fatigue measured by the FSS did not fit the data well. Therefore, academicians could conduct further research on the use of the FSS (IRT) proposed in this study and when it is appropriate to use the FSS.

### Limitations and future study

There are a few limitations to this study. First, the mean value of the FSS was high in the current sample, and the peak probability of response in each category was biased toward respondents with higher ability. This study was conducted during the spread of COVID-19 infection in Japan, which affected people’s daily lives. Although the Japanese government did not implement strong restrictions (e.g., lockdowns), it did implement intermittent activity restrictions; that is, the bias in the peak response probability may be due to the COVID-19 pandemic and the related changes in society, such as isolation and social distancing practices. Therefore, the conclusions about the measurement performance of the FSS presented herein are made in the context of the impact of this pandemic-related stress. For example, it may be that the spread of COVID-19 affected how people experienced stress and fatigue and how much people restricted their behaviors. Future studies should account for social situations that could be related to fatigue.

Second, the intraclass correlation of the FSS was not high (0.59), but the internal consistency (Cronbach’s alpha) was high (0.94). This may indicate that the FSS measures the temporal aspect of fatigue, which can vary over an 18-day measurement interval. However, as fatigue is a symptom of depression and the period considered for assessing depression symptoms is about 14 days, the FSS may not provide a stable measure for assessing the association between different symptoms of depression. Furthermore, the characteristics of the FSS may be responsible for the smaller regression coefficients compared to those of the MFI. Future research could examine the relationship between the temporal characteristics of the FSS and various mental health problems, including depression.

Third, environmental stress status and sleep duration were evaluated by asking only one question each. For stress status, the question asked whether physical or mental stress was “high” and did not measure the intensity of that stress. Regarding sleep duration, it has recently been pointed out that measures such as social jetlag are also correlated with depression [38], which highlights the need to collect a wide range of data on sleep habits, including bedtime and waking time during the weekdays and weekend, to clarify the relationship between these measures and depression. As this study focused on two fatigue-related scales, the FSS and MFI, such a wide range of sleep data was not measured. Future researchers could further probe into the relationship between sleeping habits and fatigue.

## Electronic supplementary material

Below is the link to the electronic supplementary material.

Supplementary Material 1

## Data Availability

The datasets generated during and/or analyzed during the current study are available from the corresponding author on reasonable request.

## List of Abbreviations

CTT: Classical Test Theory
DSM-5: Diagnostic and Statistical Manual of Mental Disorders – 5th edition
FSS: Fatigue Severity Scale
GRM: Graded Response Model
ICC: Item Characteristic Curve
IIC: Item Information Curves
ISI: Insomnia Severity Index
IRT: Item Response Theory
MFI: Multidimensional Fatigue Inventory
MnSq: Mean Square Statistics
PHQ-9: Patient Health Questionnaire
TIF: Test Information Function
VIF: Variance Inflation Factor
