# Supplementary material for: Evaluating the psychometric properties of the fatigue severity scale using item response theory
Source: BMC Psychol. 2023 May 12;11:155. doi: 10.1186/s40359-023-01198-z (PMC10177705; doi:10.1186/s40359-023-01198-z)
Supplement: Supplementary file 1 — Supplementary Material 1 [file 40359_2023_1198_MOESM1_ESM.docx]

Table S1. Multiple comparison of the Multidimensional Fatigue Inventory, Fatigue Severity Scale, and Patient Health Questionnaire-9 by environmental stress status groups

|  | Group 1  (n = 176) | Group 2  (n = 47) | Group 3  (n = 93) | Group 4  (n = 307) | Multiple comparison  (Tukey’s honestly significant difference test) |
| --- | --- | --- | --- | --- | --- |
| MFI | 59.32 | 55.72 | 59.69 | 51.52 | 4 < 1, 4 < 3 |
|  | (11.75) | (8.85) | (11.39) | (12.53) |  |
| General fatigue | 13.18 | 12.77 | 13.30 | 10.69 | 4 < 1, 4 < 2, 4 < 3 |
|  | (3.13) | (2.55) | (2.96) | (3.24) |  |
| Physical fatigue | 11.94 | 10.94 | 11.89 | 9.89 | 4 < 1, 4 < 3 |
|  | (3.15) | (2.61) | (3.06) | (3.29) |  |
| Reduced activation | 10.86 | 10.28 | 10.91 | 10.11 | 4 < 1 |
|  | (2.96) | (2.56) | (2.98) | (3.11) |  |
| Reduced motivation | 11.65 | 10.89 | 11.43 | 10.47 | 4 < 1, 4 < 3 |
|  | (2.73) | (2.31) | (2.53) | (2.87) |  |
| Mental fatigue | 11.70 | 10.85 | 12.15 | 10.37 | 4 < 1, 4 < 3, 2 < 3 |
|  | (2.53) | (1.93) | (2.62) | (2.78) |  |
| FSS | 3.56 | 3.29 | 3.74 | 2.83 | 4 < 1, 4 < 3 |
|  | (1.25) | (1.13) | (1.25) | (1.25) |  |
| PHQ-9 | 6.07 | 3.94 | 7.46 | 3.12 | 4 < 1, 2< 1, 2 < 3, 4 < 3 |
|  | (5.58) | (3.91) | (6.36) | (3.88) |  |

Note. N = 623. The number in parentheses is the standard deviation. FSS: Fatigue Severity Scale, MFI: Multidimensional Fatigue Inventory, PHQ-9: Patient Health Questionnaire-9. Group 1: mentally stressful environment (work or school), Group 2: physically stressful environment (work or school), Group 3: mentally and physically stressful environment (work or school), Group 4: non-stressful environment (work or school).

Table S2. 95% confidence interval of multiple comparisons of the Multidimensional Fatigue Inventory, Fatigue Severity Scale, and Patient Health Questionnaire-9

|  | Groups | 95% CI lower | 95% CI upper |
| --- | --- | --- | --- |
| MFI | 4-1 | -10.71 | -4.90 |
|  | 4-3 | -11.81 | -4.53 |
| General fatigue | 4-1 | -3.25 | -1.73 |
|  | 4-2 | -3.34 | -0.81 |
|  | 4-3 | -3.57 | -1.66 |
| Physical fatigue | 4-1 | -2.83 | -1.28 |
|  | 4-3 | -2.97 | -1.04 |
| Reduced activation | 4-1 | -1.48 | -0.16 |
| Reduced motivation | 4-1 | -1.85 | -0.51 |
|  | 4-3 | -1.80 | -0.13 |
| Mental fatigue | 4-1 | -1.97 | -0.68 |
|  | 4-3 | -2.58 | -0.97 |
| FSS | 4-1 | -1.03 | -0.43 |
|  | 4-3 | -1.29 | -0.53 |
| PHQ-9 | 2-1 | -4.18 | -0.08 |
|  | 4-1 | -4.12 | -1.76 |
|  | 3-2 | 1.28 | 5.76 |
|  | 4-3 | -5.82 | -2.86 |

Note. 95% CI, 95% confidence interval, FSS: Fatigue Severity Scale, MFI: Multidimensional Fatigue Inventory, PHQ-9: Patient Health Questionnaire-9.

Table S3. Multiple comparison of the Multidimensional Fatigue Inventory, Fatigue Severity Scale, and Patient Health Questionnaire-9 by sleep duration groups

|  | Group 1  (n = 16) | Group 2  (n = 79) | Group 3  (n = 189) | Group 4  (n = 198) | Group 5  (n = 110) | Group 6  (n = 24) | Group 7  (n = 7) | Multiple comparison  (Tukey’s honestly significant difference test) |
| --- | --- | --- | --- | --- | --- | --- | --- | --- |
| MFI | 66.06 | 60.53 | 54.48 | 53.64 | 53.72 | 55.46 | 61.71 | 1 > {3,4,5}, 2 > {3,4,5} |
|  | (8.55) | (11.54) | (12.28) | (11.85) | (12.85) | (12.31) | (17.75) |  |
| General fatigue | 14.75 | 13.29 | 11.97 | 11.51 | 11.28 | 11.50 | 13.29 | 1 > {3,4,5,6}, 2 > {3,4,5} |
|  | (3.42) | (3.40) | (3.29) | (3.07) | (3.46) | (2.97) | (4.03) |  |
| Physical fatigue | 13.69 | 12.18 | 10.56 | 10.47 | 10.49 | 11.00 | 12.71 | 1 > {3,4,5}, 2 > {3,4,5} |
|  | (3.06) | (3.28) | (3.20) | (3.13) | (3.31) | (3.73) | (3.69) |  |
| Reduced activation | 12.00 | 11.48 | 10.15 | 10.10 | 10.36 | 11.29 | 11.86 | 2 > {3,4} |
|  | (1.94) | (2.87) | (2.96) | (2.81) | (3.33) | (3.40) | (3.72) |  |
| Reduced motivation | 12.88 | 11.67 | 10.84 | 10.84 | 10.63 | 10.88 | 12.14 | 1 > 5 |
|  | (3.10) | (2.80) | (2.77) | (2.65) | (2.89) | (2.47) | (2.59) |  |
| Mental fatigue | 12.75 | 11.91 | 10.96 | 10.71 | 10.95 | 10.79 | 11.71 | 2>4 |
|  | (2.68) | (2.26) | (2.84) | (2.61) | (2.78) | (2.40) | (4.30) |  |
| FSS | 4.16 | 3.41 | 3.07 | 3.08 | 3.30 | 3.46 | 3.65 | 1>{3,4} |
|  | (1.18) | (1.20) | (1.22) | (1.27) | (1.44) | (1.20) | (1.89) |  |
| PHQ-9 | 9.63 | 6.78 | 4.81 | 3.93 | 3.48 | 3.75 | 7.86 | 1>{3,4,5}, 2>{4,5} |
|  | (6.86) | (5.83) | (5.06) | (4.40) | (4.41) | (5.07) | (7.92) |  |

Note. MFI: Multidimensional Fatigue Inventory, FSS: Fatigue Severity Scale, PHQ-9: Patient Health Questionnaire-9. Group 1: sleep duration less than 4 hours, Group 2: between 4–5 hours, Group 3: 5–6 hours, Group 4: 6–7 hours, Group 5: 7–8 hours, Group 6: 8–9 hours, Group 7: 9 hours or more.

Table S4. 95% confidence interval of multiple comparison of Multidimensional Fatigue Inventory, Fatigue Severity Scale, and Patient Health Questionnaire-9 among sleep duration groups

|  | Groups | 95% CI lower | 95% CI upper |  | Groups | 95% CI lower | 95% CI upper |
| --- | --- | --- | --- | --- | --- | --- | --- |
| MFI | 3-1 | -21.00 | -2.17 | Reduced activation | 3-2 | -2.51 | -0.14 |
|  | 4-1 | -21.82 | -3.03 |  | 4-2 | -2.56 | -0.2 |
|  | 5-1 | -22.02 | -2.67 | Reduced motivation | 5-1 | -4.44 | -0.05 |
|  | 3-2 | -10.90 | -1.21 | Mental fatigue | 4-2 | -2.26 | -0.13 |
|  | 4-2 | -11.71 | -2.08 | FSS | 3-1 | -2.09 | -0.10 |
|  | 5-2 | -12.15 | -1.48 |  | 4-1 | -2.07 | -0.09 |
| General fatigue | 3-1 | -5.31 | -0.25 | PHQ-9 | 3-1 | -8.65 | -0.97 |
|  | 4-1 | -5.77 | -0.71 |  | 4-1 | -9.53 | -1.86 |
|  | 5-1 | -6.07 | -0.87 |  | 5-1 | -10.09 | -2.20 |
|  | 6-1 | -6.39 | -0.11 |  | 4-2 | -10.63 | -1.12 |
|  | 3-2 |  |  |  | 5-2 | -4.82 | -0.89 |
|  | 4-2 | -2.63 | -0.02 |  |  |  |  |
|  | 5-2 | -3.07 | -0.49 |  |  |  |  |
| Physical fatigue | 3-1 | -5.63 | -0.62 |  |  |  |  |
|  | 4-1 | -5.72 | -0.72 |  |  |  |  |
|  | 5-1 | -5.77 | -0.62 |  |  |  |  |
|  | 3-2 | -2.9 | -0.33 |  |  |  |  |
|  | 4-2 | -2.99 | -0.43 |  |  |  |  |
|  | 5-2 | -3.1 | -0.27 |  |  |  |  |

Note. FSS: Fatigue Severity Scale, MFI: Multidimensional Fatigue Inventory, PHQ-9: Patient Health Questionnaire-9.

| Item 1 | Item 2 | Item 3 |
| --- | --- | --- |
| 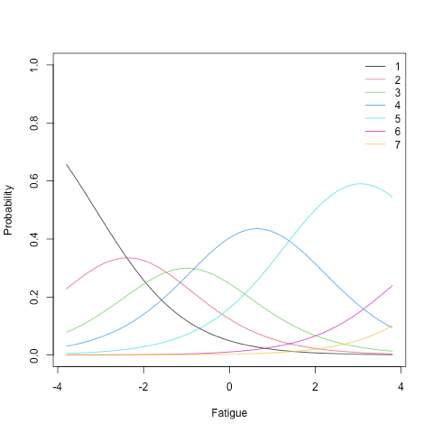 | 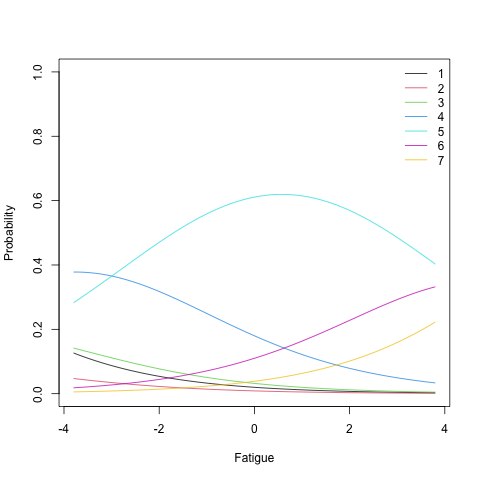 | 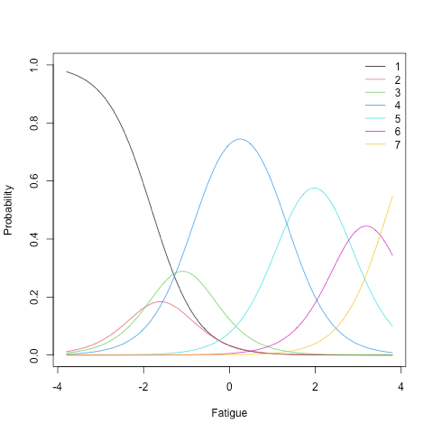 |
| Item 4 | Item 5 | Item 6 |
| 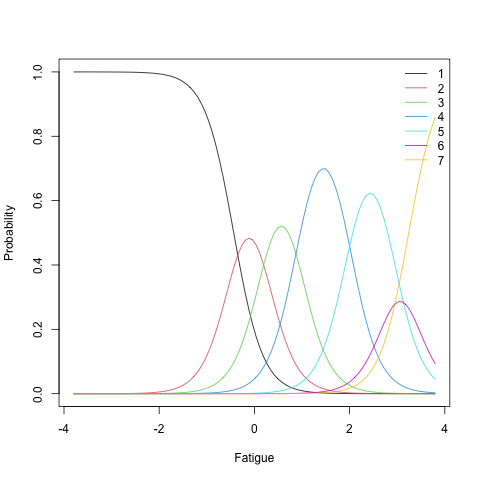 | 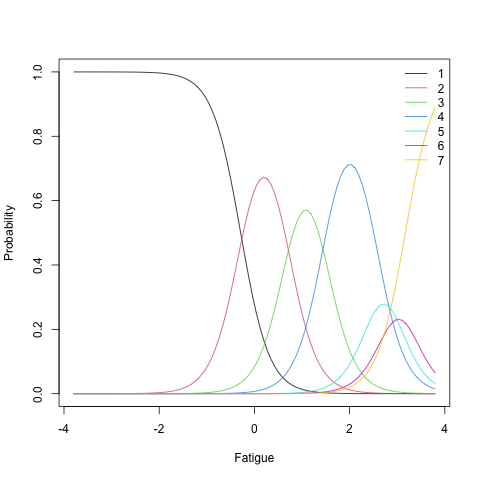 | 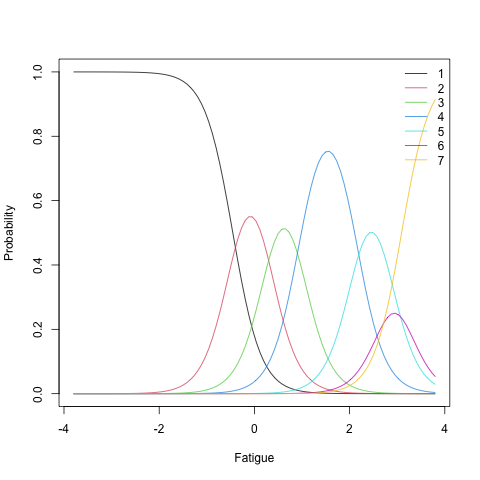 |
| Item 7 | Item 8 | Item 9 |
| 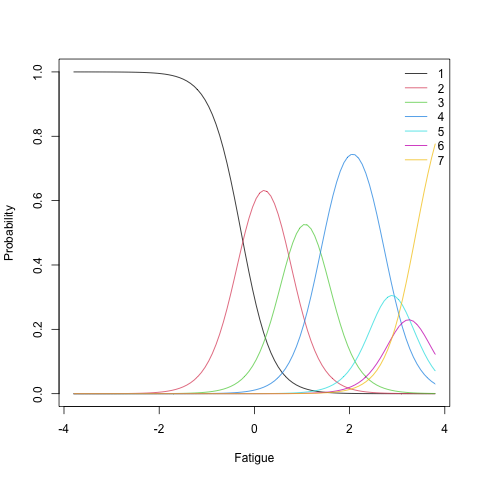 | 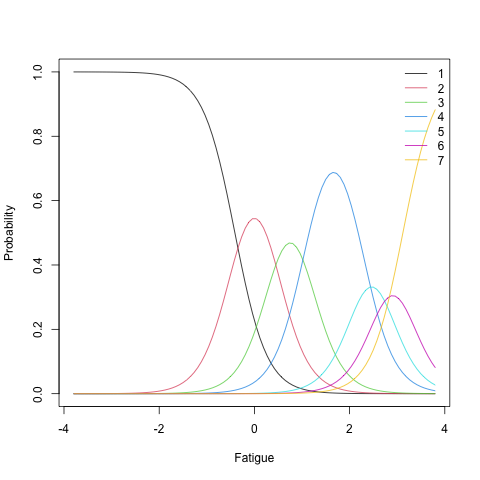 | 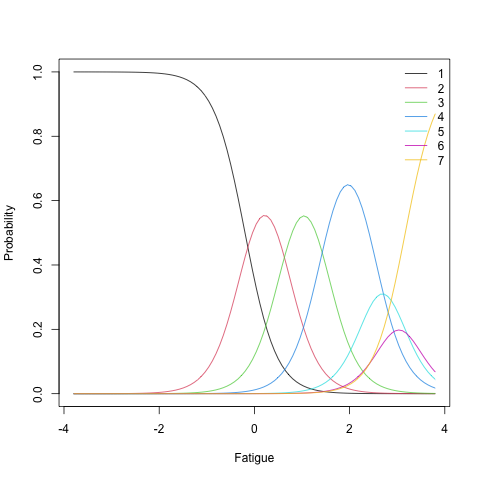 |
| IIC | TIF |  |
| 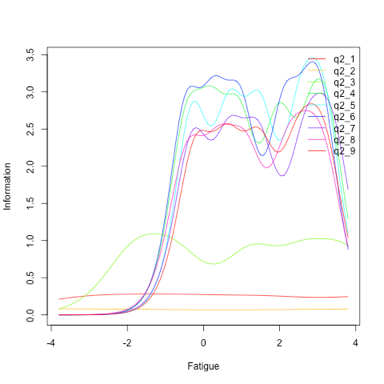 | 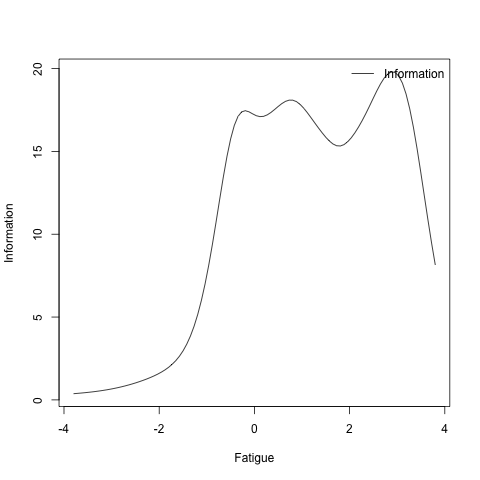 |  |

**Figure S1. Item characteristic curve, item information curve, test information function of Condition 0.** In Condition 0, the original Fatigue Severity Scale was used. Eigenvalues: 5.85, 1.18, 0.44, 0.39, 0.35. ICC: item characteristic curve, IIC: item information curve, TIF: test information function.

| Item 3 | Item 4 | Item 5 |
| --- | --- | --- |
| 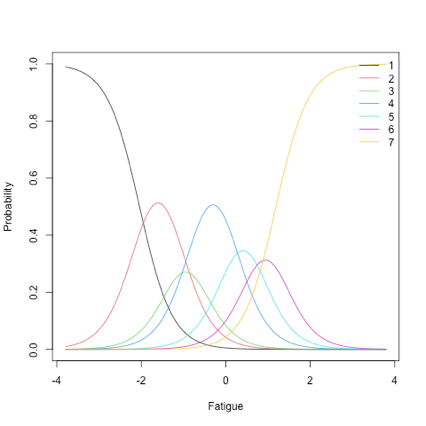 | 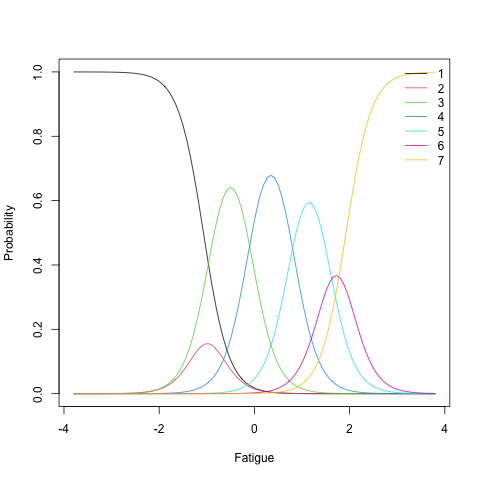 | 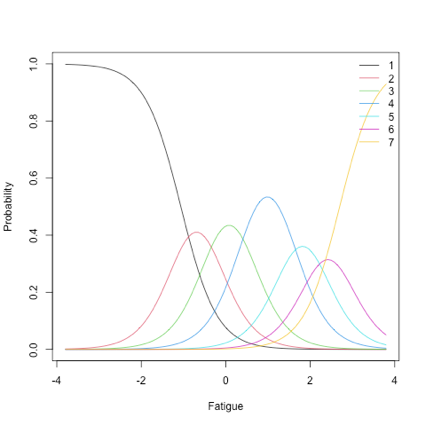 |
| Item 6 | Item 7 | Item 8 |
| 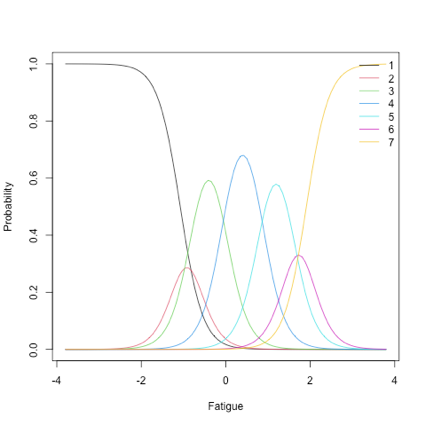 | 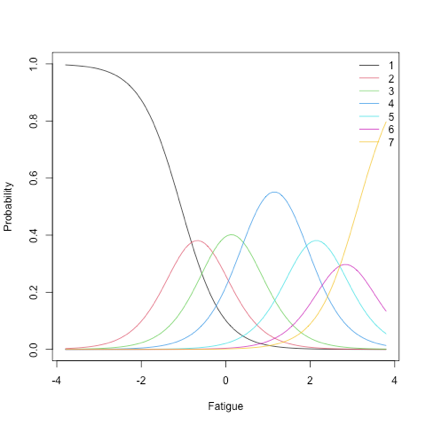 | 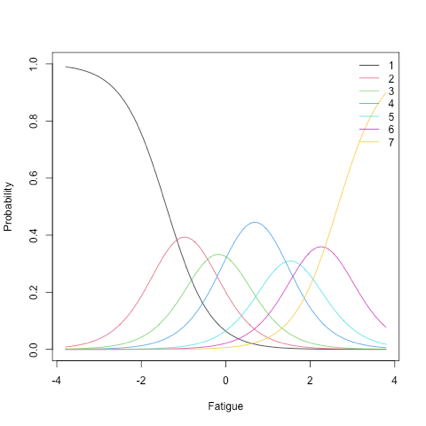 |
| Item 9 | IIC | TIF |
| 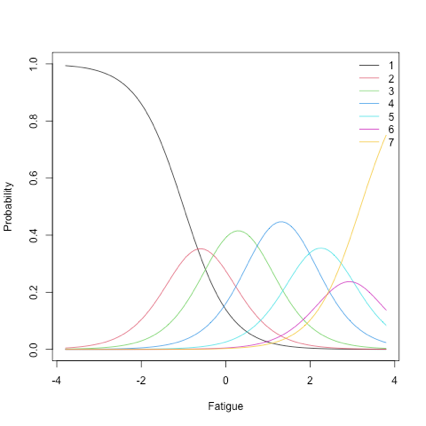 | 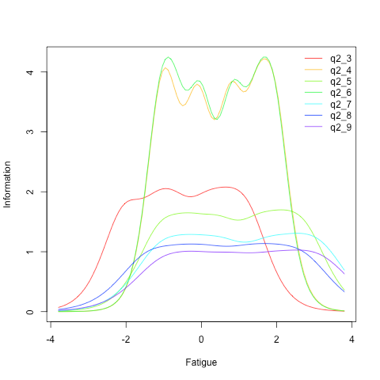 | 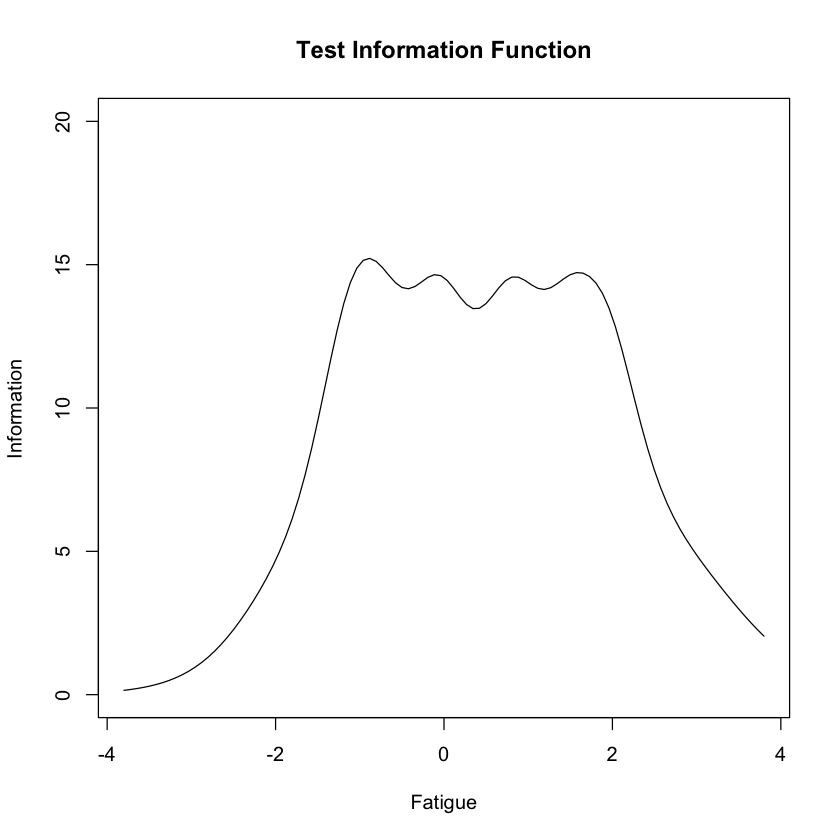 |

**Figure S2. Item characteristic curve, item information curve, test information function of Condition 1.** In Condition 1, items 1 and 2 of the Fatigue Severity Scale were removed. Eigenvalues: 5.16, 0.61, 0.42, 0.23. ICC: item characteristic curve, IIC: item information curve, TIF: test information function.

| Item 1 | Item 2 | Item 3 |
| --- | --- | --- |
| 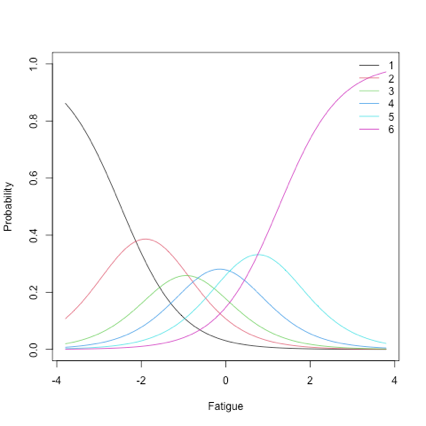 | 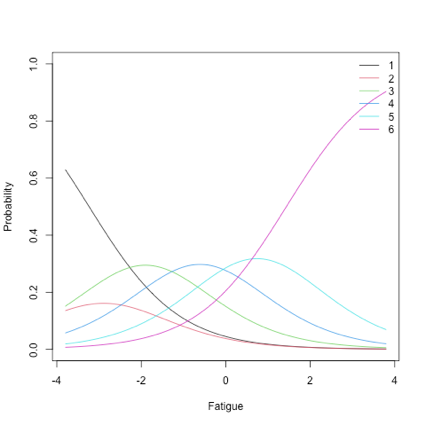 | 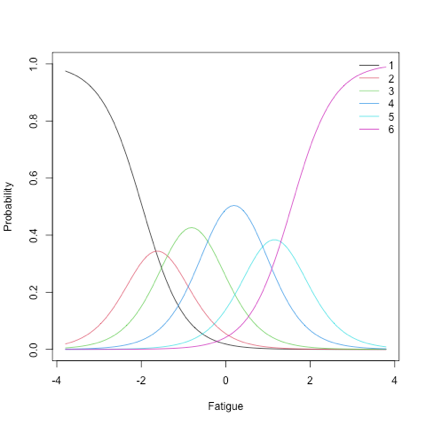 |
| Item 4 | Item 5 | Item 6 |
| 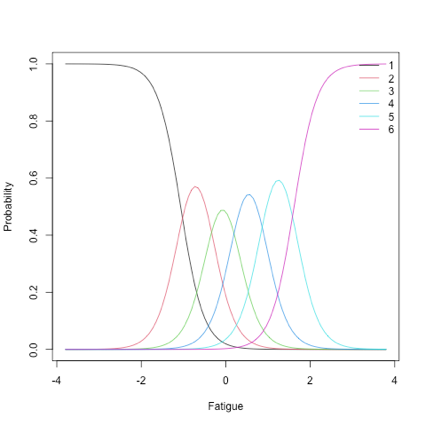 | 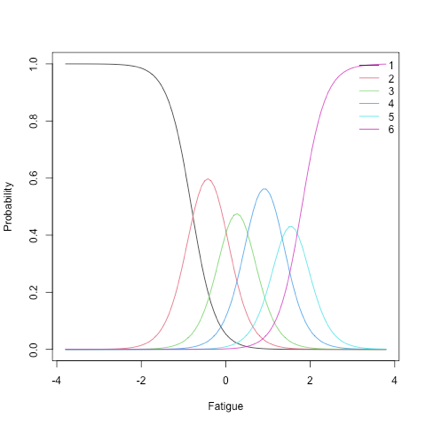 | 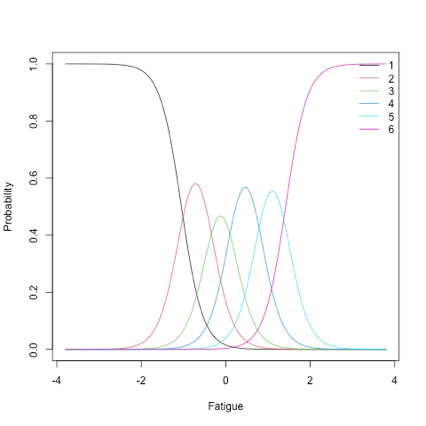 |
| Item 7 | Item 8 | Item 9 |
| 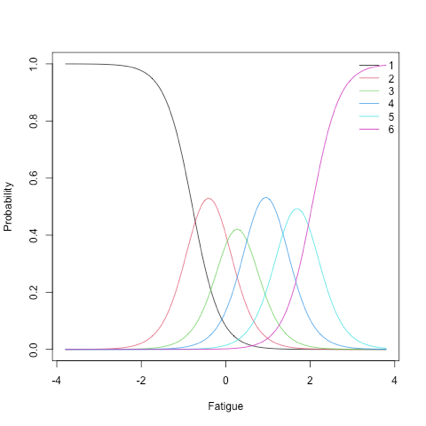 | 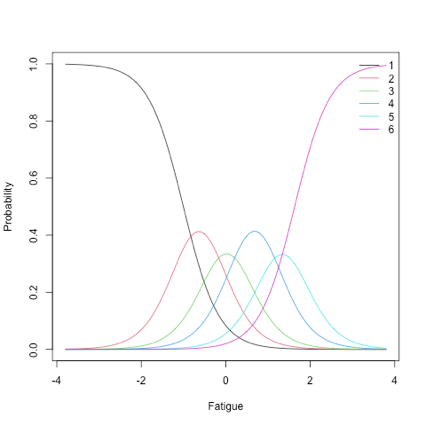 | 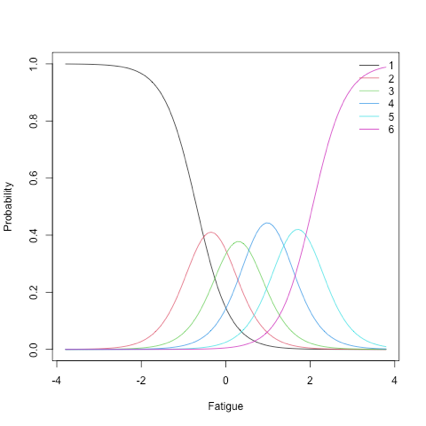 |
| IIC | TIF |  |
| 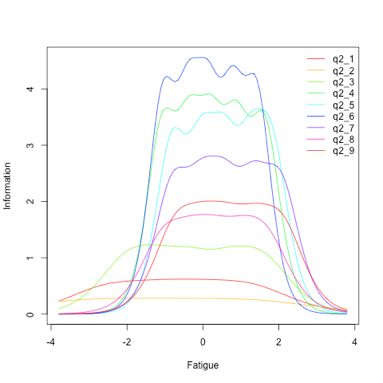 | 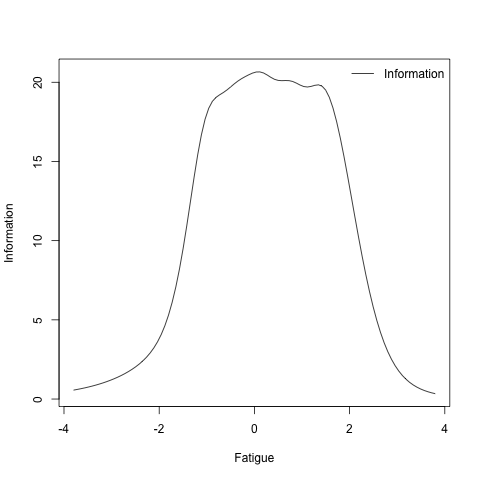 |  |

**Figure S3. Item characteristic curve, item information curve, test information function of Condition 2.** In Condition 2, Grades 6 and 7 of the Fatigue Severity Scale were integrated. Eigenvalues: 5.80, 1.19, 0.45, 0.41. ICC: item characteristic curve, IIC: item information curve, TIF: test information function.

| Item 3 | Item 4 | Item 5 |
| --- | --- | --- |
| 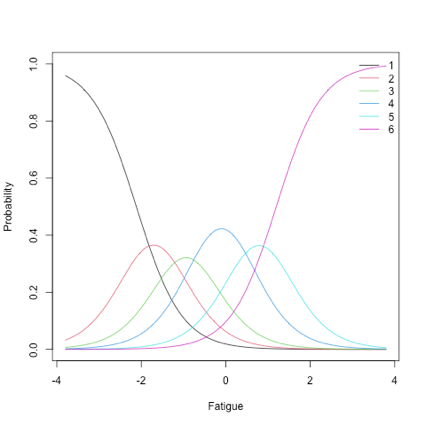 | 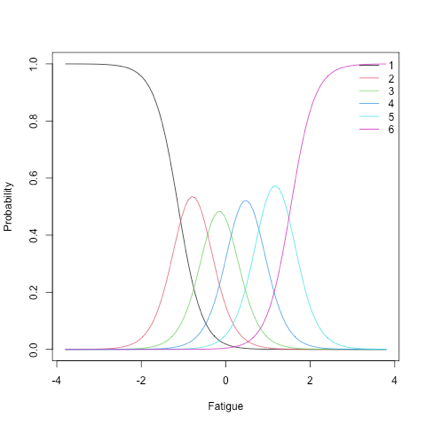 | 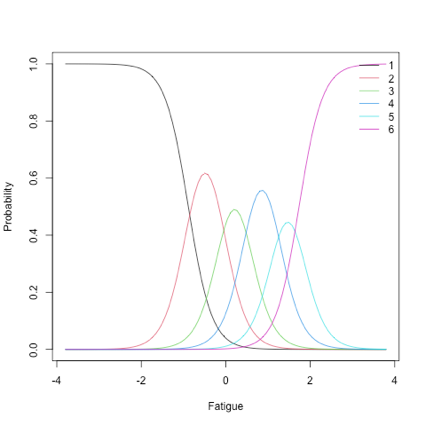 |
| Item 6 | Item 7 | Item 8 |
| 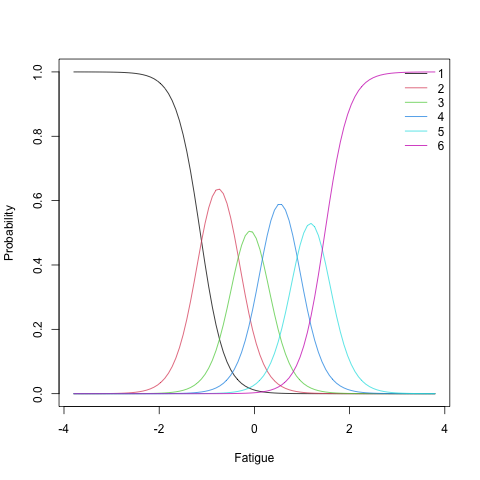 | 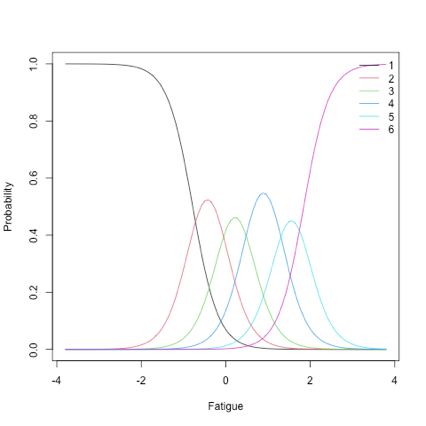 | 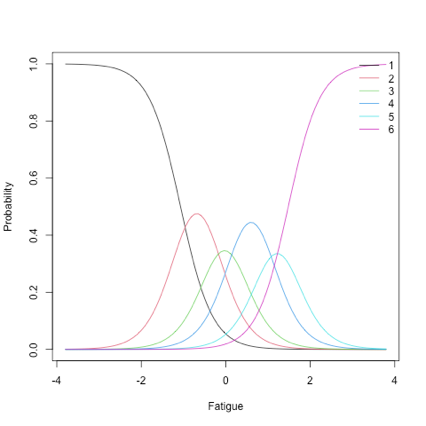 |
| Item 9 | IIC | TIF |
| 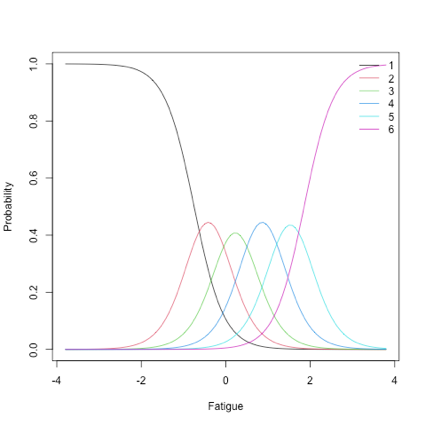 | 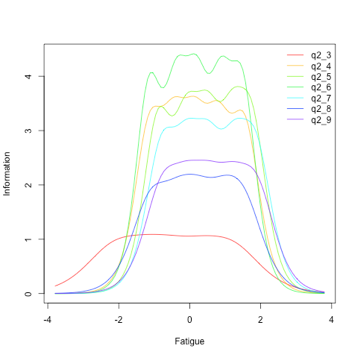 | 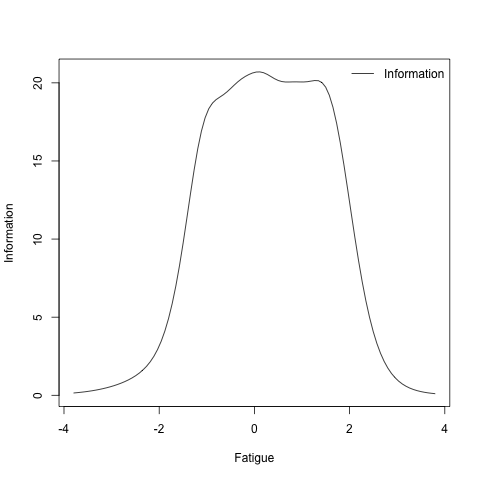 |

**Figure S4. Item characteristic curve, item information curve, test information function of Condition 3.** In Condition 3, items 1 and 2 of the Fatigue Severity Scale were removed and Grades 6 and 7 were integrated. Eigenvalues: 5.12, 0.62, 0.43, 0.24. ICC: item characteristic curve, IIC: item information curve, TIF: test information function.

| 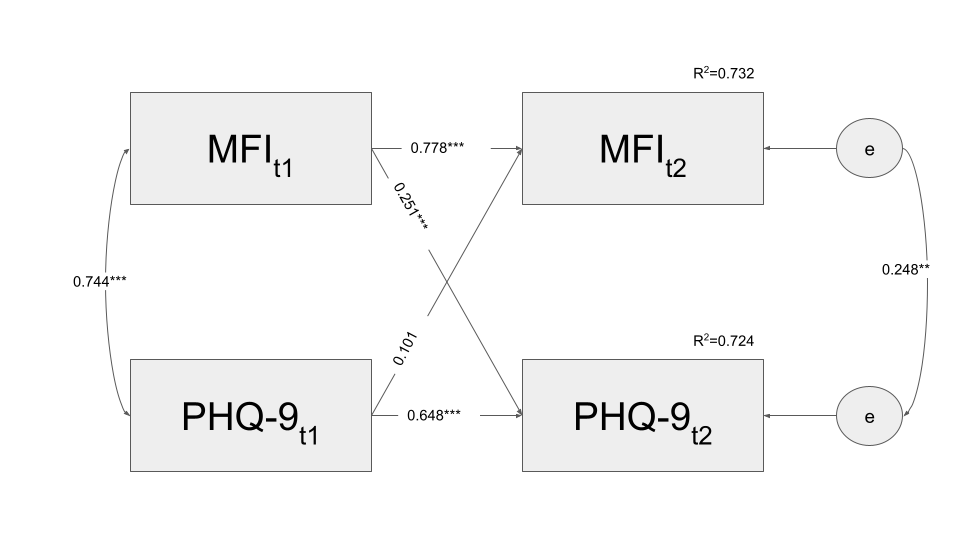 | 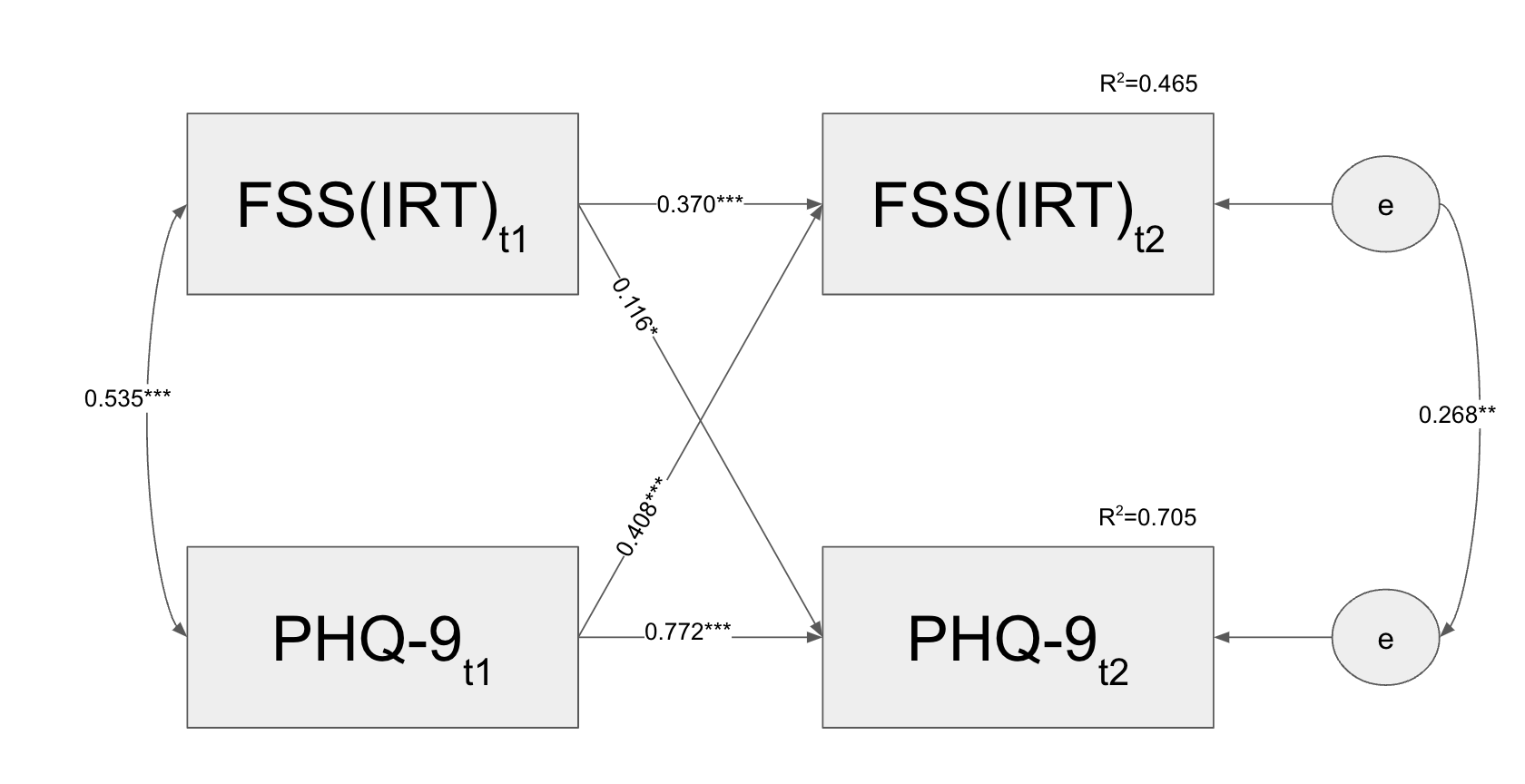 |
| --- | --- |
| (a) The association between MFI and PHQ-9 | (b) The association between FSS and PHQ-9 |

**Figure S5. Results for the synchronous effects model.** Note. N = 125. MFI: Multidimensional Fatigue Inventory, FSS: Fatigue Severity Scale, PHQ-9: Patient Health Questionnaire, t1: Time 1, t2: Time 2. *** *p* < .001.
